# Piperacillin/tazobactam Induced Myelosuppression

**DOI:** 10.4021/jocmr2009.03.1227

**Published:** 2009-03-24

**Authors:** Kirsty Wai Chung Lee, Kai Ming Chow, Natalie Pui Ha Chan, Angeline Oi Shan Lo, Cheuk Chun Szeto

**Affiliations:** a Department of Medicine & Therapeutics, Prince of Wales Hospital, The Chinese University of Hong Kong, Shatin, Hong Kong, China; b Department of Anatomical & Cellular Pathology, Prince of Wales Hospital, The Chinese University of Hong Kong, Shatin, Hong Kong, China

## Abstract

Neutropenia, secondary to immune destruction or maturation arrest, is the most commonly described adverse haematological effect of beta-lactam antibiotics. We describe a case of reversible pancytopenia, with evidence of hypocellular marrow, after a prolonged course of piperacillin/tazobactam for 26 days.

**Keywords:**

Piperacillin; Tazobactam; Myelosuppression; Neutropenia

## Introduction

Piperacillin/tazobactam is a semisynthetic ureidopenicillin and a beta-lactamase inhibitor, with broad-spectrum and increased activity against Pseudomonas aeruginosa when compared with other penicillins. Bone marrow suppression is a recognized adverse drug reaction of beta-lactam antibiotics. A recent systematic review have supported the assumption that piperacillin can also have such adverse effects [1]. We report a case of reversible bone marrow toxicity associated with piperacillin/tazobactam use. This report reinforces previous suggestions that monitoring of haematological parameters is crucial in patients receiving prolonged treatment with piperacillin.

## Case Report

**Figure 1:**
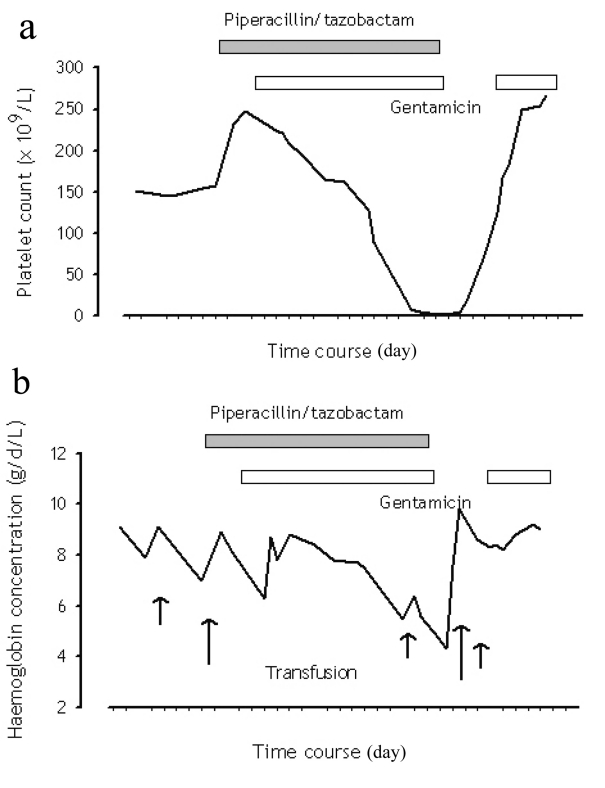
Trend of (a) platelet count and (b) haemoglobin concentration in relation to piperacillin/tazobactam administration.

**Figure 2:**
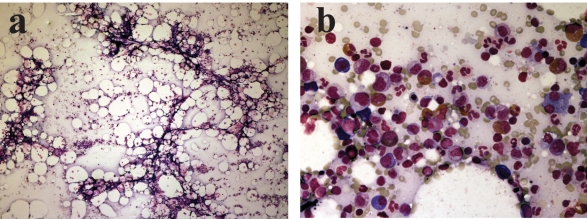
(a) Very hypocellular marrow aspirate at low power view; (b) Cellular area from the hypocellular bone marrow aspirate showing granulopoiesis with markedly reduced erythropoiesis.

A 60-year-old man with a history of failed renal transplant on peritoneal dialysis was admitted for parainfluenza infection. He had a recent history of video-assisted thoracoscopic surgery with pleurodesis for right pleural effusion four months ago. On admission, he was noted to have a temperature of 38^o^C and a right chest wall abscess over the previous surgical site. Computed tomography of the thorax showed loculated pleural collections and subsequent culture of pleural aspirate grew Pseudomonas aeruginosa. The patient was started on piperacillin/tazobactam (2/0.25 g every 8 hours) and gentamicin. Complete blood count at baseline showed chronic hypochromic normocytic anemia (haemoglobin 89 g/L), platelet count 232 x 10^9^/L and white cell count 10.5 x 10^9^/L. After 23 days of antimicrobial therapy, our patient developed thrombocytopenia of 128 x 10^9^/L, whilst haemoglobin level decreased to 77 g/L. On day 26 of treatment, his platelet count was further reduced to 90 x 10^9^/L. Both piperacillin/tazobactam and gentamicin was stopped after 30 days of piperacillin/tazobactam treatment, in view of possible bone marrow toxicity. Two days after stopping the antibiotic, haemoglobin level measured 55 g/L, and the platelet count 9 x 10^9^/L. The lowest haemoglobin level and platelet count were 43 g/L and 2 x 10^9^/L respectively (Fig. 1); transfusion support was given. Bone marrow examination (Fig. 2a, b) then demonstrated a hypocellular marrow characterized by markedly reduced erythropoiesis and megakaryopoiesis, without abnormal infiltration. Parvovirus B19-specific IgM antibody and polymerase chain reaction assay were both negative.

His haemoglobin level and platelet count recovered after stopping piperacillin/tazobactam. His empyema was subsequently treated with cefoperazon/sulbactam and gentamicin for another three weeks, with no further cytopenia observed.

## Discussion

Reversible myelosuppression has been reported as an uncommon adverse reaction of piperacillin. Like other beta-lactams, neutropenia is the most common abnormality, and is often associated with mild thrombocytopenia. Thrombocytopenia rarely occurs alone, and isolated anaemia has not been described before.

The mechanism by which marrow toxicity develops remains speculative. It is believed that piperacillin causes proliferation arrest of the myeloid cells. Only one case report of piperacillin/tazobactam related pancytopenia had been documented by bone marrow aspiration [2], which demonstrated a rich marrow with maturation arrest of all lineages. Moreover, published studies have also detected immunologically-mediated haemolytic anaemia and thrombocytopenia [1], in the presence of IgG antibodies against penicillins. Despite recent attention in the adverse haematological effects of piperacillin, current understanding is by and large derived from sporadic case reports pertaining to leucopenia [3-5], anaemia or thrombocytopenia [5-8].

More large-scale cohort studies suggested that the myelotoxicity is related to large cumulative doses. The adverse haematological reaction rarely occurs with treatment less than 10 days, although a study of 41 patients receiving piperacillin/tazobactam for bone-related infections showed no significant difference between the mean treatment duration of neutropenic and non-neutropenic patients [3]. The same study demonstrated a significantly younger mean age for the group with neutropenia. Younger patients, especially children, tend to be more vulnerable. Of the three published case reports regarding this topic, the patients were between the ages of 18 to 27 years [2, 6, 7]. In a case series of 25 patients, including five subjects under the age of 18, piperacillin was used to treat Pseudomonas infection. Six patients subsequently developed neutropenia, of which four were children [5]. Children appear to develop haematological toxicity with a shorter duration of piperacillin therapy when compared to adults. One case report has suggested that this effect may be related to body weight [2], and recommends a dosage reduction in underweight patients.

Although piperacillin/tazobactam is eliminated in the urine, patients with renal insufficiency receiving dose-adjusted piperacillin/tazobactam therapy have not been reported to be at increased risk of myelotoxicity. Nevertheless, the delayed recovery of pancytopenia in our patient may well be explained by underlying renal failure, as well as the presence of hypocellular marrow, both of which have been found to be adverse prognostic factors for recovery in toxic neutropenia [9]. The true incidence of piperacillin bone marrow suppression is hitherto unknown, and may be explained by a paucity of data involving more than two weeks of piperacillin/tazobactam therapy. Much less is known about the genetic predisposition to this haematological adverse reaction of piperacillin/tazobactam. Further studies will also be critical in determining whether and, if so, which signal heralds the development of bone marrow suppression after piperacillin/tazobactam.

The key points from this case are: Piperacillin/tazobactam treatment, if prolonged, can be associated with bone marrow suppression; Besides the oft-quoted neutropenia, other manifestation includes thrombocytopenia and/or pancytopenia; Vigilance by clinicians in detecting and diagnosing this reversible adverse drug reaction is important; Early recognition should probably be achieved by regular monitoring of blood counts in patients receiving more than 10 days of piperacillin/tazobactam.

In conclusion, cytopenia involving any of the myeloid lineages may occur with prolonged piperacillin/tazobactam treatment. It would be wise to recommend, pending further evidence on this haematological adverse reaction of piperacillin, regular monitoring of blood counts in patients receiving more than 10 days of therapy.
